# Freeze-Dried Extracellular Vesicles From Adipose-Derived Stem Cells Prevent Hypoxia-Induced Muscle Cell Injury

**DOI:** 10.3389/fcell.2020.00181

**Published:** 2020-03-20

**Authors:** Khairat Bahgat Youssef El Baradie, Mohamed Nouh, Frederick O’Brien III, Yutao Liu, Sadanand Fulzele, Ali Eroglu, Mark W. Hamrick

**Affiliations:** ^1^Medical College of Georgia, Augusta University, Augusta, GA, United States; ^2^Faculty of Science, Tanta University, Tanta, Egypt; ^3^Tanta Cancer Center, Tanta, Egypt; ^4^Dwight D. Eisenhower Army Medical Center, Fort Gordon, Augusta, GA, United States

**Keywords:** exosome, ultrafiltration, ultracentrifugation, hypoxia, trehalose, PVP

## Abstract

Cellular therapies have tremendous potential for the successful treatment of major extremity wounds in the combat setting, however, the challenges associated with transplanting stem cells in the prolonged field care (PFC) environment are a critical barrier to progress in treating such injuries. These challenges include not only production and storage but also transport and handling issues. Our goal is to develop a new strategy utilizing extracellular vesicles (EVs) secreted by stem cells that can resolve many of these issues and prevent ischemic tissue injury. While EVs can be preserved by freezing or lyophilization, both processes result in decrease in their bioactivity. Here, we describe optimized procedures for EVs production, isolation, and lyophilization from primary human adipose-derived stem cells (hADSCs). We compared two isolation approaches that were ultrafiltration (UF) using a tangential fluid filtration (TFF) system and differential ultracentrifugation (UC). We also optimized EVs lyophilization in conjunction with trehalose and polyvinylpyrrolidone 40 (PVP40) as lyoprotectants. Bioactivity of EVs was assessed based on reversal of hypoxia-induced muscle cell injury. To this end, primary human myoblasts were subjected to hypoxic conditions for 6 h, and then treated with hADSC-derived EVs at a concentration of 50 μg/mL. Subsequently, muscle cell viability and toxicity were evaluated using MTS and LDH assays, respectively. Overall, nanoparticle tracking data indicated that UF/TFF yields threefold more particles than UC. Lyophilization of EVs resulted in a significantly reduced number of particles, which could be attenuated by adding lyoprotections to the freeze-drying solution. Furthermore, EVs isolated by UF/TFF and freeze-dried in the presence of trehalose significantly increased viability (*P* < 0.0193). Taken together, our findings suggest that the isolation and preservation methods presented in this study may enhance therapeutic applications of EVs.

## Introduction

The field of regenerative medicine has produced significant and innovative discoveries related to the use of stem cells to heal human tissue (Pati et al., 2015). A number of studies support the use of mesenchymal stem cells (MSCs), induced pluripotent stem cells (iPSCs), and embryonic stem cells (ESCs) to enhance tissue repair following injury (Caplan, 2007; Gimble et al., 2007; Hirschi et al., 2014). The primary mechanism of action by which these cells are thought to improve tissue healing is via the release of secreted factors (e.g., growth factors and cytokines) that function in a paracrine manner (Caplan and Dennis, 2006). Indeed, it is estimated that 80% of the therapeutic effect of stem cells is mediated through paracrine actions of the stem cell “secretome” (Uccelli et al., 2008; Maguire, 2013). It has recently been found that stem cells secrete high levels of EVs, generally referred to as exosomes and microvesicles. Exosomes are small (<150 nm) and microvesicles are larger (>150 nm) membrane-bound particles that carry proteins, microRNAs (miRNAs), and mRNAs (Hodges et al., 2017; Murphy et al., 2018). These nanoparticles are endocytosed by recipient cells, and thus are key players in cell-to-cell communication (Thery, 2011). Importantly, stem cell-derived EVs can promote tissue repair in a variety of organs and tissues (Tetta et al., 2012). For example, EVs produced by MSCs improve cell survival in the heart and kidney following ischemia-reperfusion injury (Arslan et al., 2013; Chen et al., 2013) as well as in the fetal brain after hypoxia-ischemia (Ophelders et al., 2016) and EVs can also support peripheral nerve regeneration (Ching and Kingham, 2015).

It is thought that stem cell-derived EVs ameliorate ischemia-reperfusion injury primarily by delivering enzymes that increase ATP production and activate survival pathways in recipient cells (Arslan et al., 2013). EVs also promote angiogenesis in target tissues by transferring specific microRNAs such as miR-125a (Liang et al., 2016) as well as angiogenic proteins such as vascular endothelial growth factor (VEGF) (Franquesa et al., 2014). MSCs are the most common cell type used for EVs production, and these include both adipose- and bone-derived MSCs. Adipose-derived MSCs are generally more abundant than bone-derived MSCs (Gimble et al., 2007), and both adipose-derived MSCs and their secreted EVs demonstrate no immunogenicity in allergenic or even xenogenic transplantation (Lin et al., 2012), underscoring their therapeutic potential. While cellular therapies have tremendous potential for tissue repair, an alternative strategy is to utilize the stem cell secretome itself to promote cell survival and regeneration. Indeed, EVs have demonstrated their effectiveness at improving cutaneous wound healing (Hu et al., 2016) and skin flap ischemia-reperfusion injury (Bai et al., 2018). Limb ischemia is major challenge in the trauma setting, and prolonged ischemia can lead to skeletal muscle death (Blaisdell, 2002). The application of stem cell-derived EVs to the preservation of skeletal muscle in the ischemic environment may therefore represent one approach for tissue salvage in the trauma setting.

Typically, the differential ultracentrifugation (UC) method has been used for isolation of EVs (Jeppesen et al., 2014). More recently, the ultrafiltration (UF) method using a tangential fluid filtration (TFF) system proved to be effective in isolating EVs from large fluid volumes (Andriolo et al., 2018; Busatto et al., 2018). Although the isolated EVs can be frozen or lyophilized for long-term storage, both approaches are known to decrease the bioactivity of EVs by compromising their integrity to different extents (Bari et al., 2018). Thus, there is a need for optimized methods for cryopreservation and lyophilization of EVs. In particular, successful lyophilization of EVs could represent an “off-the-shelf” strategy for utilizing the stem cell secretome in a therapeutic context (Ge et al., 2014). Here, we test two hypotheses that (1) the UF using TFF system is more efficient than the UC method at isolating exosomes from hADSCs and (2) such EVs can maintain their effectiveness for promoting skeletal muscle cell survival under hypoxic conditions when freeze-dried by using a rationally designed lyophilization approach.

## Materials and Methods

### Culture of Human Adipose-Derived Stem Cells

StemPro^TM^ Human Adipose-Derived Stem Cells (hADSCs) were purchased from Thermo Fisher Scientific (Catalog # R7788115; Norcross, GA, United States). The cells were cultured in monolayer conditions in Dulbecco’s Modified Eagle Medium (DMEM/High Glucose; Hyclone, Logan, UT, United States), supplemented with 5% (v/v) fetal bovine serum (FBS; Atlanta Biologicals, Atlanta, GA, United States) and 1% (v/v) penicillin/streptomycin (Pen-Strep; Gibco, Grand Island, NY, United States). The hADSCs were cultured at 37°C under 5% CO_2_ and used at passages 5–6 in all experiments.

### Preparation of Conditioned Medium and Isolation of hADSC-Exosomes

hADSCs were seeded in 10/150 mm dishes within DMEM/High Glucose, supplemented with 5% (v/v) FBS and 1% (v/v) penicillin/streptomycin. Once the hADSCs reached a sub-confluence state of ∼80%, they were first washed with the culture medium containing 5% exosome-depleted FBS, and then cultured in the same medium (25 mL/dish) for 24 h. Next, the conditioned medium was collected from each dish and 25 mL of fresh medium was added to each dish for another 24 h of culture. At the end of the culture period, the conditioned medium was collected from each dish again and mixed together with previously collected one to obtain a total of 500 mL conditioned medium.

The half (250 mL) of the conditioned medium was used to isolate EVs by differential UC as described by Jeppesen et al. (2014). In brief, cell debris were pelleted at 300 × g for 10 min and 2000 × g for 20 min. Larger vesicles were pelleted at 10,000 × g for 30 min, and the supernatant was filtered through a 0.2 μm Nalgene PES membrane filter (Thermo Fisher Scientific, Waltham, MA, United States). Next, exosomes were pelleted at 100,000 × g for 120 min using 70 mL polycarbonate tubes (355622; Beckman Coulter, Brea, CA, United States). The exosome pellets were resuspended either in 250 μL of sterile phosphate buffer saline (PBS; Hyclone, Logan, UT, United States) or in 250 μL of 50 mM trehalose (Trehalose dihydrate; Sigma-Aldrich, St. Louis, MD, United States) to use in different experiments.

The remaining half (250 mL) of the conditioned medium was subjected to TFF for isolation of exosomes. Cell debris and larger vesicles were pelleted at 10,000 × g for 30 min, the supernatant filtered through a 0.2 μm PES membrane filter. The conditioned medium was then subjected to ultrafiltration in a Labscale Tangential Flow Filtration System (TFF; EMD Millipore Corporation, Billerica, MA, United States) using 300 kDa Pellicon XL Cassette. A feed flow rate of 30–50 mL/min, transmembrane pressure < 3.5 psi, and crossflow rate > 10:1 were maintained throughout the filtration process. Once the conditioned medium was concentrated to 10-fold, we started the diafiltration step via changing the media with the 5X volume of 1X PBS. The final product was sterile filtered using a 0.2 μm filter, resulting in a filtrate that contains exosomes in 1X PBS for the subsequent step.

### Testing the Effectiveness of Exosome-Isolation Methods

This set of experiments was designed to compare the effectiveness of two methods (i.e., UC and UF/TFF) in isolating EVs (exosomes) by evaluating their size, number, and bioactivity. In addition, 50 mM trehalose prepared in 1X PBS was tested as a suspension buffer and lyoprotectant with respect to 1X PBS. Thus, the main test groups were (1) UC, (2) UF/TFF, and (3) UF/TFF plus lyophilization whereas the subgroups included (a) fresh EVs suspended 1X PBS and (b) exosomes suspended in 50 mM trehalose. For the latter, the buffer was exchanged with 5X volume of 50 mM trehalose at the diafiltration step. The resulting product contained exosomes suspended in sterile 50 mM trehalose/1X PBS. The EVs suspended in 50 mM trehalose and allocated to Group 3 (UF/TFF + Lyophilization) were first frozen at −80°C overnight, and then lyophilized using the 2.5 L −50°C Benchtop Freeze Dryer (Labconco Corporation, Kansas, MO, United States). The lyophilized EV samples were stored at room temperature until experimental testing. They were rehydrated with ddH_2_O by adding the original volume, and then vortexed before using for NTA and bioactivity assays along with the EVs from other two groups as described below.

### Optimization of Exosome Lyophilization

For this set of experiments, the UF/TFF method was used to isolate EVs based on the outcome of the first set of experiments. To further optimize the lyophilization process in terms of preserving bioactivity of EVs, we tested different concentrations of trehalose and PVP40 as lyoprotectants. To maintain the overall osmolality of the lyophilization solutions at around 290 mOsm/kg, the strength of PBS was adjusted according to trehalose concentrations while PVP40 concentrations were ignored due to their insignificant contribution to the overall osmolality (Table 1). For the first group, 0.095 g trehalose was dissolved in sterile 4 ml of 0.83X PBS, and then 1 mL of freshly prepared exosomes in 1X PBS were added to bring the total volume to 5 mL. The final concentration of the trehalose was 50 mM. For the second group, the same approach was used with a modification that included adding 0.12 g PVP40. The resulting final concentrations of trehalose and PVP40 were 50 mM and 2.5%, respectively. For the third group, the concentration of PVP40 increased to 5% while the trehalose concentration remained unchanged at 50 mM. The same strategy was used to prepare additional three different combinations of trehalose and PVP40, which included 100 mM trehalose + 0% PVP40, 100 mM trehalose + 2.5% PVP40, and 100 mM trehalose + 5% PVP40. To prepare the 100 mM trehalose, 0.191 g of trehalose was dissolved in sterile 4 ml of 0.65X PBS, then 1 mL of freshly prepared EVs in 1XPBS were added to bring the total volume to 5 mL (Table 1).

**TABLE 1:** Experimental groups and preparation of freeze-drying solutions containing exosomes.

Experimental groups	Trehalose added	PVP40 added	Volume and strength of PBS	Exosomes added	Total volume
1X PBS (Control)	0.000 g	0.000 g	4 mL of 1X PBS	1 mL in 1X PBS	5 mL
50 mM Trehalose + 0.0% PVP40	0.095 g	0.000 g	4 mL of 0.83X PBS	1 mL in 1X PBS	5 mL
50 mM Trehalose + 2.5% PVP40	0.095 g	0.125 g	4 mL of 0.83X PBS	1 mL in 1X PBS	5 mL
50 mM Trehalose + 5.0% PVP40	0.095 g	0.250 g	4 mL of 0.83X PBS	1 mL in 1X PBS	5 mL
100 mM Trehalose + 0.0% PVP40	0.191 g	0.000 g	4 mL of 0.65X PBS	1 mL in 1X PBS	5 mL
100 mM Trehalose + 2.5% PVP40	0.191 g	0.125 g	4 mL of 0.65X PBS	1 mL in 1X PBS	5 mL
100 mM Trehalose + 5.0% PVP40	0.191 g	0.250 g	4 mL of 0.65X PBS	1 mL in 1X PBS	5 mL

*To maintain the osmolality around 290 mOsm/kg, diluted PBS solutions at 0.83X and 0.65X strengths were used for preparation of 50 and 100 mM trehalose whereas PVP’s contribution is assumed to be negligible. To ensure the sterility, freeze-drying solutions containing trehalose and PVP were prepared in larger volumes and then sterile filtered before adding exosomes.*

For the exposure experiments, the EVs were next held in their respective freeze-drying solutions at 4°C for 1 h, and then trehalose and PVP40 were removed by diafiltration using the TFF system with 1X PBS. Subsequently, NTA and bioactivity evaluation were performed.

For the freeze-drying experiments, the 5 ml of each EVs samples prepared in the aforementioned freeze-drying solutions were divided into 10 polypropylene microcentrifuge tubes, each tube contains 0.5 ml of lyophilization solution supplied with exosomes. After the EVs samples were placed in safe-lock polypropylene microcentrifuge tubes and were frozen overnight at -80 degrees. Immediately before loading into the freeze dryer, the tubes were opened, and a film in which 7 holes were pierced (1 mm diameter each) was placed on top of the tube’s opening. The samples were loaded into 2.5 L −50°C Benchtop Freeze Dryer (Labconco Corporation, Kansas, 150 MO, United States), which has a condenser temperature as low as – 55°C and a vacuum pump capable of reaching an absolute pressure of 0.039 mBar. Samples were lyophilized for 10 h (with the environmental temperature conditioned to 22°C) and were stored at room temperature (23–25°C) in the tightly sealed box to prevent moisture absorption and light exposure. Before their use, the lyophilized EVs were rehydrated with ddH_2_O by adding the original volume and vortexed subsequently. Next, trehalose and PVP40 were removed by diafiltration using the TFF system with 1XPBS, and subsequently, NTA and bioactivity evaluation were performed.

### Nanoparticle Tracking Analysis (NTA)

NTA was performed using the ZetaView PMX 110 (Particle Metrix, Meerbusch, Germany) and its corresponding software (ZetaView 8.04.02). Samples were diluted 1:100 in PBS, manually injected into the instrument. The instrument measured each sample at 11 different positions throughout the cell, with two cycles of readings at each position. After automated analysis of all 11 positions and removal of any outlier positions, the mean, median, and mode (indicated as diameter) sizes, as well as the concentration of the sample, were calculated by the optimized machine software. For each measurement, the instrument pre-acquisition parameters were set to a temperature of 23°C, a sensitivity of 85, a frame rate of 15 frames per second (fps), a shutter speed of 100, and a laser pulse duration equal to that of shutter duration. The number of particles per particle size curves was created using quadratic interpolation. The average of three samples was taken in order to compare the data.

### Western Blotting

First, protein concentration was measured using Synergy H1 Multi-Mode Reader (BioTek, Winooski, VT, United States) in each sample. Next, 4X Laemmli buffer was added to exosomes samples containing 50 μg/μL protein. The EVs samples were then loaded onto 4–20% sodium dodecyl sulfate-polyacrylamide gel, subjected to electrophoresis, and transferred to a polyvinylidene fluoride transfer membrane, followed by blocking for 1 h. The membrane was incubated with the following antibodies: anti-CD63 (1:200 dilution), anti-CD81 (1:200 dilution), anti-TSG 101 (1:200 dilution), anti-ToMM20 (1:200 dilution) and anti-APOA1/2 (1:200 dilution) overnight at 4°C. The membrane was then incubated with horseradish peroxidase (HRP)-conjugated goat anti-rabbit IgG (1:1000 dilution) for 1 h at room temperature. The membrane was reacted with Immobilon Western Chemiluminescent HRP substrate, and chemiluminescence was detected and quantified using an infrared Odyssey machine (LI-COR Biosciences). Because UC samples had low protein contents (Table 2), larger sample volumes were loaded to achieve a protein concentration similar to that of UF samples. The low protein content of the UC samples may be a reflection of their lower EVs yield.

**TABLE 2:** Protein content of hADSc cell lysate, UC, UF, and UF/lyophilization exosomes.

Sample	Protein yield mean ± SD (μg/μL)
hADSCs Cell lysate	5.3±0.01
UF/Treh.	3.8±0.02
UF/Lyo Treh.	3.5±0.012
UC/Treh.	2±0.013
UF/1XPBS	3.9±0.023
UF/Lyo 1XPBS	2±0.03
UC/1XPBS	1.9±0.01

### Transmission Electron Microscopy

For initial fixation, EVs samples were mixed with an equal volume of 4% paraformaldehyde in PBS for 1 h. The fixed samples were applied to carbon Formvar film-coated transmission electron microscopy (TEM) grid, incubated for 20 min at room temperature, and then were wicked off with filter paper. After washing with PBS, the samples were fixed with 1% glutaraldehyde for 5 min. Upon washing with distilled water, the grid was stained with 1% uranyl acetate for 30 s, wicked off with Whatmen filter paper, and then allowed to dry before viewing. TEM examination was performed using JEM 1230 transmission electron microscope (JEOL USA Inc., Peabody, MA, United States) at 110 kV and imaged with an UltraScan 4000 CCD camera and First Light Digital Camera Controller (Gatan Inc., Pleasanton, CA, United States). The preparation and imaging of TEM samples were performed at the Electron Microscopy and Histology Core Laboratory at Augusta University^1^.

### Hypoxic Cell Culture and Treatment of Primary Human Myoblasts

It has been shown that exposure of myoblast monolayers to nutrition depletion and hypoxia for 6–12 h can simulate certain characteristics of skeletal muscle ischemia, which include a significant increase in partial pressures of oxygen (PO2) decreases in carbon dioxide (PCO2) and a pH of the extracellular fluid. The overall effect of these changes is increasing the percentage of apoptotic cells, which is a feature of skeletal muscle ischemia. We hypothesized that stem cell-derived EVs ameliorate ischemia-reperfusion injury and activate survival pathways in recipient cells (Arslan et al., 2013). To test this hypothesis, we performed the following steps: Primary Human Skeletal Myoblasts purchased from Thermo Fisher Scientific (Catalog # A11440; Norcross, GA, United States) were cultured as monolayers in DMEM/High Glucose, supplemented with 5%(v/v) FBS and 1% (v/v) penicillin/streptomycin. The medium was changed after 24 h to discard non-adherent cells. Media were changed every 2 days, and the cells were expanded until passage 4–6. The expanded myoblast cells were then seeded in 48-well plates at density 7000cells/well and allowed to attach and grow for 24 h. Next day, the culture medium was changed to DMEM containing 4.5 g/L glucose without L-glutamine and Phenol red (Corning, Manassas, VA, United States), and the myoblasts were exposed to hypoxia condition (1% O_2_ and 5% CO_2_) for 6 h. At the end of the hypoxia period, exosomes from different experimental groups (Table 1) were added to the injured myoblasts at a concentration of 50 μg/μL. For the control group, the ischemic myoblasts were treated with freshly prepared EVs (50 μg/μL) in 1X PBS.

We also ran parallel experiments where ischemic myoblasts were treated either with phenol red-free DMEM supplemented with 1% FBS as a control group or with different concentrations of sodium hydrosulfide NaHS or sodium hydrosulfide donor (GYY4137, Cayman Chemical), since hydrogen sulfide has previously been shown to attenuate ischemic injury (Henderson et al., 2011; Wetzel and Wenke, 2019) we were interested in comparing the efficiency of exosomes treatment to other treatment approaches.

A stock solution of sodium hydrosulfide (NaHS; Cayman Chemical, Ann Arbor, MI, United States) was prepared by dissolving it in PBS at a concentration of 10 mM. Next, different volumes of the stock solution were added to myoblast cultures during the last 20 min of the hypoxia-period to obtain 100 and 10 μM final concentrations of NaHS in the culture medium (phenol red-free DMEM supplemented with 1% FBS). At the end of the hypoxia period, the myoblast cultures were transferred to a normoxic incubator for additional 3 h. To test the effect of the H_2_S donor, a stock solution of GYY4137 was prepared in PBS and added to myoblast cultures at the end of the hypoxia period to obtain different final concentrations of 100 and 10 μM. After treatment under the normoxic condition for 24 h, the myoblasts were subjected to bioactivity assays (MTS and LDH, see below).

### Assaying Viability and Proliferation of Ischemic Primary Human Myoblasts

To determine if freeze-dried EVs and NaHS can ameliorate the ischemic injury in a similar manner, ischemic human myoblasts were first treated with EVs and NaHS or its donor as explained above, and then subjected to both MTS and LDH assays according to the manufacturer’s protocol. Briefly, the ischemic myoblasts in each well were mixed with 100 μL of 3-(4,5-dimethylthiazol-2-yl)-5-(3-carboxymethoxyphenyl)-2-(4-sulfophenyl)-2H-tetrazolium, inner salt (MTS, Promega Corporation, FL, United States) after removal of the culture medium at the end of the normoxic incubation period while a group without cells served as the blank. The optical density (OD) was measured at 490 nm using a microplate reader. Each group was run in sextuplicate. For the LDH assay, media from each treatment group were collected at the end of the normoxic incubation period, and the absorbance was measured at 490 nm and 680 nm. The LDH activity was determined after the subtraction of the 680nm absorbance value (background) from the 490nm absorbance. The percentage of cytotoxicity was calculated as% Cytotoxicity = (Compound-treated LDH activity–spontaneous LDH Activity)/(Maximum LDH activity–spontaneous LDH activity) × 100. The data for each group were obtained from sextuplicate experiments and shown as mean percentages.

### Statistical Analysis

All data sets obtained were expressed as means ± SD. Data were tested for statistical significance by using Graph-Pad Prism 8 software. GraphPad Prism 8 was utilized to perform ANOVA with Bonferroni pairwise comparison or unpaired *t*-tests as appropriate. A *p*-value of 0.05 or less was considered significant.

## Results

### Characterization of hADSC-Derived EVs Obtained via UC, UF, and UF/Lyophilization (UF/LYO)

**FIGURE 1:**
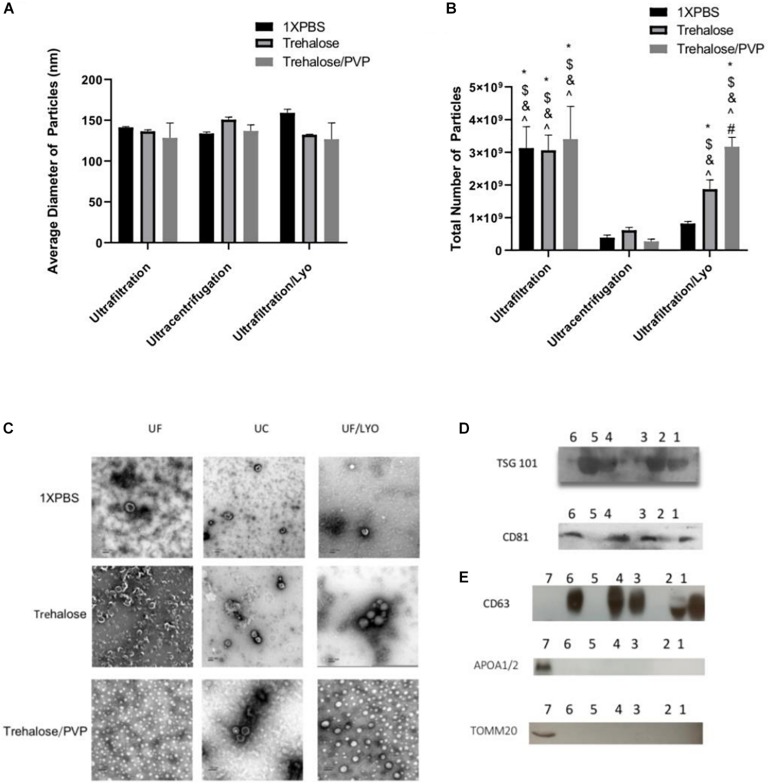
Identification and characterization of exosomes secreted by human ADSCs. Exosomes were isolated either by Ultracentrifugation (UC), Ultrafiltration (UF) or Ultrafiltration/Lyophilization (UF/LYO) from supernatants of hADSCs using 1XPBS, 100 mM Trehalose (Tre) and 100 mM Trehalose + 5% PVP, the lyophilized exosomes were rehydrated with ddH_2_O by adding the original volume and vortexed immediately before use. NTA using the ZetaView instrument was done to measure particles diameters (nm) **(A)** and to determine the total number of particles isolated using different techniques **(B)**. Purified exosomal pellets after negative staining display a pure population exosomes with typical morphology **(C)** while Western blotting shows the protein level of the exosomal markers TSG101, and CD81 for different experimental groups **(D)**, Lane 1 = UF/Tre; Lane 2 = UF/LYO-Tre; Lane 3 = UC/Tre; Lane 4 = UF/1X PBS; Lane 5 = UF/LYO-1XPBS; Lane 6 = UC/1XPBS. For CD63 and for the negative marker of the purity and the specificity of the small EVs apolipoproteins A1/2 (anti-APOA1/2) anti-ToMM20 **(E)**. 1 = UF/1XPBS, 2 = UF/Tre + Lyo. 3 = UC/Tre. 4 = UF/1XPBS, 5 = UF/1XPBS + LYO, 6 = UC/1XPBS, 7 = hADSCs lysate. Data are expressed as mean ± SD (*n* = 6). For NTA total number of particles, *P < 0.0001 UF 1XPBS, 100 mM trehalose, 100 mM trehalose + 5%PVP, UF/Lyo 100 mM trehalose and 100 mM trehalose + 5% PVP vs. UC 1XPBS. ^$^*P* < 0.0001 UF 1XPBS, 100 mM trehalose, 100 mM trehalose + 5% PVP, UF/Lyo 100 mM trehalose and 100 mM trehalose vs. UC trehalose. ^&^*P* < 0.0001 UF 1XPBS, 100 mM trehalose, 100 mM trehalose + 5% PVP, UF/Lyo 100 mM trehalose and 100 mM trehalose vs. UC trehalose/PVP. ^*P* < 0.001 UF 1XPBS, 100 mM trehalose, 100 mM trehalose + 5% PVP, UF/Lyo 100 mM trehalose and 100 mM trehalose + 5% PVP vs. UF/LYO 1XPBS. ^#^*P* < 0.02 UF/Lyo 100 mM trehalose vs. UF/Lyo 100 mM trehalose + 5%PVP.

To characterize the purified exosomes derived from hADSCs via UC and UF techniques, NTA, TEM, and western blot analysis were used. NTA showed that the size of the majority of the particles was consistent with that of exosomes (50–150 nm). Furthermore, both isolation techniques yielded particles with statistically similar diameters (Figure 1A). In contrast, the total number of particles isolated with UF and UF/LYO was significantly higher than the UC at the same starting volume of 1 mL sample (Figure 1B). After confirming that the diameters of the particles are within the expected range for exosomes (50–150 nm), we further verified that these particles correspond to exosomes by using TEM imaging, a well-accepted technique for nanoparticle identification. Negatively stained exosome samples isolated with each of the three techniques showed particles with a typical cup-shaped morphology within the size range of exosomes (Figure 1C). WB analysis revealed that the particles expressed the well-established exosome biomarkers TSG 101, CD81 (Figure 1D), CD63 (Figure 1E), Additionally, the purity and the specificity of the small EVs (exosomes) have assessed using common negative control as non-integral protein, apolipoproteins A1/2 (anti-APOA1/2) and transmembrane, lipid-bound and soluble proteins associated to other intracellular compartments (mitochondria) anti-ToMM20 (Figure 1E). Overall, these results confirm that the particles collected using the aforementioned three different techniques are within acceptable quality limits in terms of size, morphology, and enrichment of biomarker proteins.

### Effectiveness of Exosomes Isolated by Different Methods in Treating Ischemic Myoblasts

**FIGURE 2:**
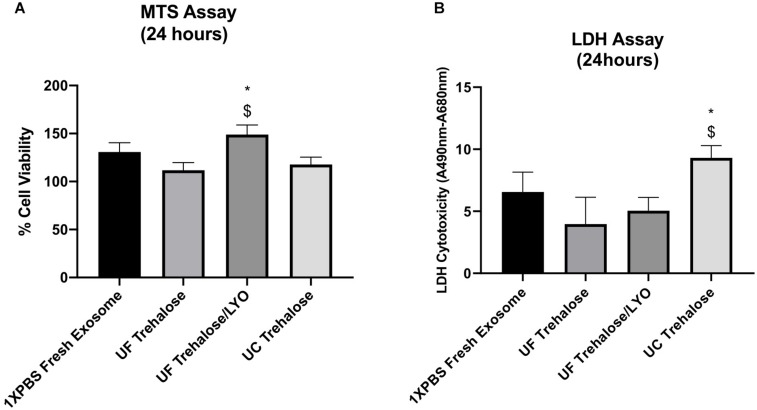
Effect of human ADSC-derived exosomes on ischemic human myoblasts. Human myoblasts were treated with hADSC-derived exosomes ultrafiltrated in 50 mM trehalose for 24 h following the exposure to hypoxia for 6 h. Shown is the effect of different techniques on proliferation of ischemic human myoblasts as determined by MTS assay **(A)** and cell viability in different groups as determined by LDH assay **(B)**. Data are expressed as mean ± SD (*n* = 6). For LDH assay, *P < 0.0262 UC trehalose vs. UF trehalose/LYo. ^$^*P* < 0.016 UC trehalose vs. UF trehalose. For MTS assay, *P < 0.0385 UC trehalose vs. UF trehalose/LYo. ^$^*P* < 0.019 UC trehalose vs. UF trehalose.

After completing the validation of hADSCs-derived exosomes purified with UC, UF and UF/LYO, we further evaluated the effects of these exosomes on the viability of ischemic human myoblasts by treating them with the same concentration (50 μg/mL) of exosomes for 24 h. MTS assay showed that there was a statistically significant difference in cell numbers between the UF/LYO group and the other two treatment (i.e., UC and UF) groups (Figure 2A). Furthermore, the LDH assay results suggest that exosomes isolated using the UC method were significantly less effective in ameliorating the ischemic injury compared to those obtained using two different UF methods (Figure 2B). Based on these results, we selected the UF method for the optimization experiments as described next.

### Optimization of Exosome Lyophilization

**FIGURE 3:**
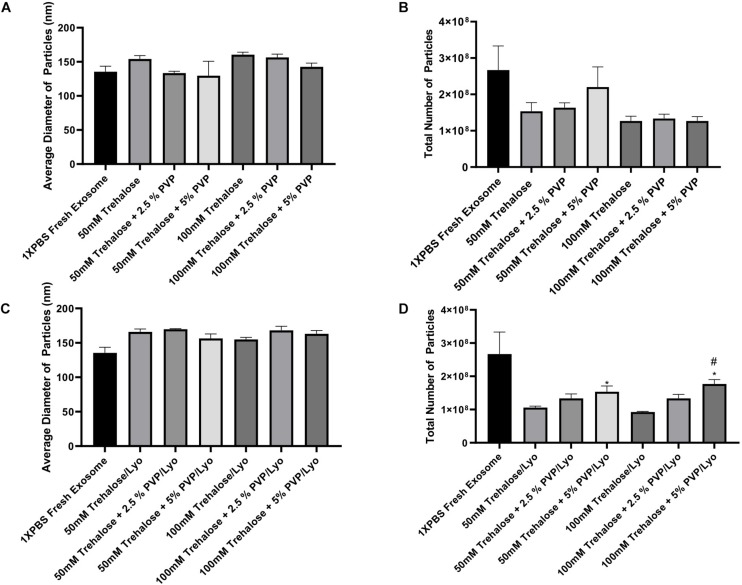
ZetaView measurements of exosomes extracted from human ADSCs using two different isolation techniques. For the exposure experiments **(A,B)**, exosomes were held in different concentrations of trehalose and PVP40 at 4°C temperature for 1 h. Then, the trehalose and PVP40 were removed by diafiltration using the TFF system with 1XPBS. For the freeze-drying experiments **(C,D)**, exosomes were lyophilized in each freeze-drying solution, stored at room temperature, rehydrated with ddH2O by adding the same starting volume, and then trehalose and PVP40 were removed by diafiltration using the TFF system with 1XPBS. Finally, NTA was performed using the ZetaView instrument to measure particles diameters **(A,C)** and to determine the total number of particles **(B,D)**. Data are expressed as mean ± SD (*n* = 6). *p < 0.0033 50 mM trehalose + 5% PVP40/LYO or 100 mM trehalose + 5% PVP40/LYO vs. 100 mM trehalose, ^#^*p* < 0.0282 100 mM trehalose + 5% PVP40/LYO vs. 100 mM trehalose + 2.5%PVP40.

To optimize the freeze-drying process, hADSC-derived exosomes obtained using the UF method were first exposed to different combinations of two lyoprotectants (i.e., trehalose and PVP40) at 4°C temperature for 1 h without undergoing the lyophilization step (Exposure experiments). Subsequently, trehalose and PVP40 were removed by diafiltration using the TFF system with 1X PBS, and then NTA of the treated exosomes were performed with respect to controls that were kept in 1X PBS. The results of NTA showed no significant difference in the diameter of individual particles exposed to different combinations of two lyoprotectants or kept in 1X PBS (Figure 3A). While the total number of particles in the experimental groups exposed to different concentrations of trehalose and PVP40 was slightly lower than that of the control group, the differences were not statistically significant (Figure 3B), suggesting that addition and removal of two lyoprotectants do not induce any significant damage to exosomes.

The next set of experiments included the lyophilization steps. Basically, exosomes isolated using the UF method were lyophilized in the presence of the same combinations of two lyoprotectants, and then NTA was performed to analyze the average diameter and total number of particles upon rehydration of the samples and removal of the lyoprotectants. The lyophilization step did also not induce any significant change in the diameter of particles (Figure 3C). Similar to the exposure experiments, the total number of particles was slightly reduced after lyophilization and rehydration steps when compared to the control group. However, combining 5% PVP40 with 50 and 100 mM trehalose yielded significantly higher numbers of particles than trehalose alone as a lyoprotectant, suggesting that combining trehalose with PVP40 better preserves exosomes (Figure 3D).

**FIGURE 4:**
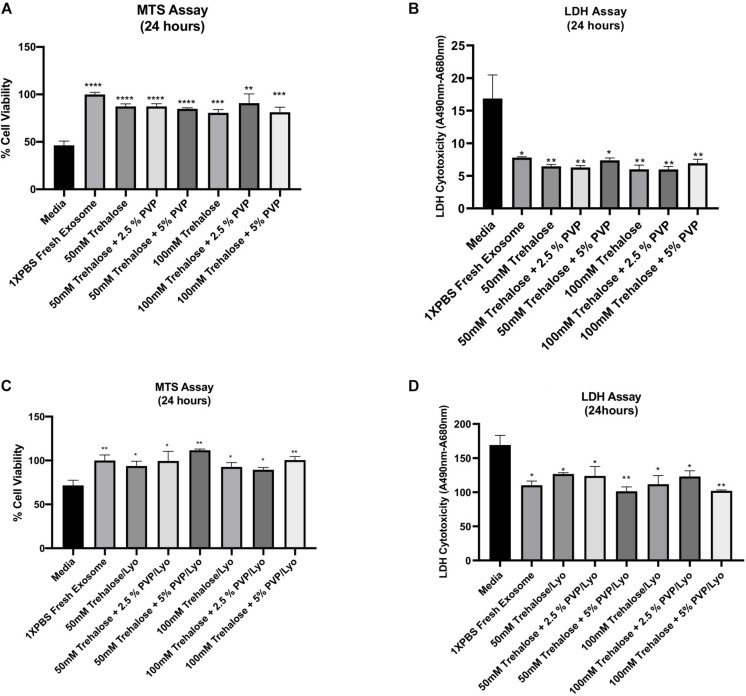
Effect of hADSC-derived exosomes on ischemic human myoblasts as analyzed by both MTS and LDH assays. For the exposure experiments **(A,B)**, hADSC-derived exosomes were collected and held in different combinations of trehalose and PVP40 at 4°C temperature for 1 h. Thereafter, trehalose and PVP40 were removed from the exosome solution by diafiltration using the TFF system with 1XPBS. Subsequently, ischemic human myoblasts were distributed to the different exposure groups and treated with the same exosome concentration (50 μg/mL) beside the media -treated group which treated with phenol red free media sublimated with 1% FBS for 24 h. For the freeze-drying experiments **(C,D)**, hADSC-derived exosomes were lyophilized in each freeze-drying solution, and then stored at ambient temperature until experimental testing. Upon rehydration and removal of trehalose and PVP40, ischemic human myoblasts treated with a 50 μg/mL concentration of the lyophilized exosomes as well as the media treated group for 24 h to determine their effects on cell proliferation and reversal of cell toxicity by MTS **(A,C)** and LDH **(B,D)** assays, respectively. Data are expressed as mean ± SD (*n* = 6). *p < 0.01, **p < 0.0056, ***p = 0.0001, ****p < 0.0001.

To verify whether the tested combinations of two lyoprotectants also preserve the bioactivity of hADSC-derived exosomes, the exposure and lyophilization experiments mentioned above were next repeated, and then the effect of the resulting exosomes on amelioration of ischemic injury was assessed by treating ischemic human myoblasts. The control groups included treatments with exosome-free medium (phenol red-free DMEM supplemented with 1% exosome-depleted FBS) and fresh exosomes suspended in 1X PBS. Except the medium control, the exosome concentration in all treatment groups was adjusted to 50 μg/mL, and the treatment duration was 24 h at 37°C. The MTS assay revealed that exposure to different combinations of two lyoprotectants does not adversely affect the beneficial bioactivity (i.e., mitigation of the ischemic injury) of the exosomes with respect to freshly prepared exosomes in 1X PBS. Compared to the medium treatment, all exosome treatment groups showed significantly higher bioactivity as assessed by the MTS assay (Figure 4A). There were no significant differences between the exosome treatment groups including the freshly prepared exosome control group. Similarly, the LDH assay showed significantly lower cell toxicity in all exosome treatment groups compared to the treatment with exosome-free medium (Figure 4B). After adding the lyophilization and rehydration steps, the results remained similar (Figures 4C,D), suggesting that the beneficial bioactivity of hADSC-derived exosomes can be preserved by freeze-drying them in the presence of trehalose and PVP40.

### Effects of NaHS and H_2_S Donor GYY4137 on Ischemic Human Myoblasts

**FIGURE 5:**
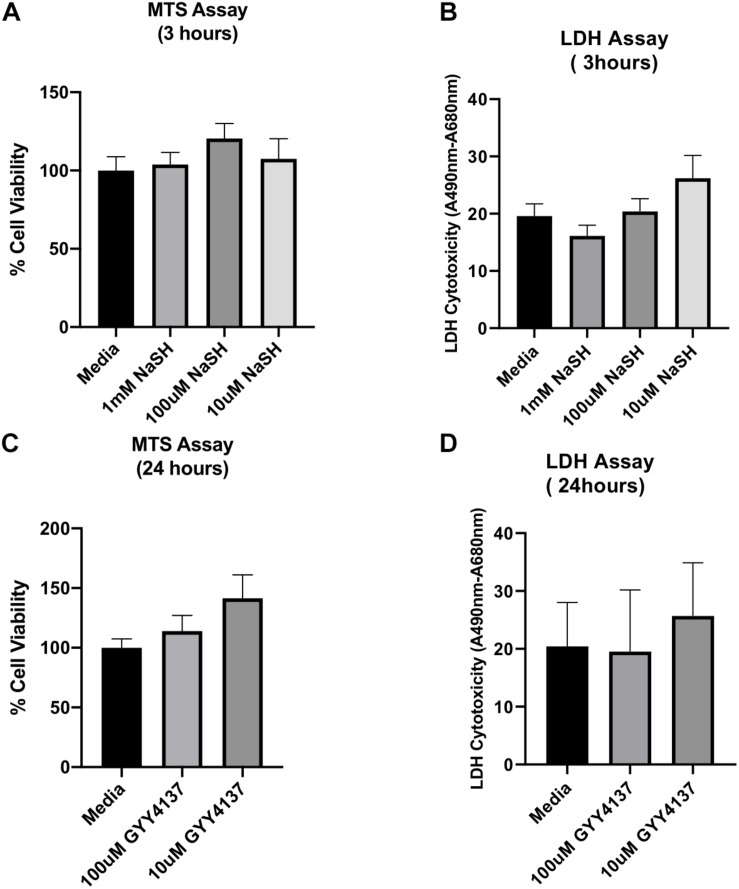
Effect of sodium hydrogen sulfide (NaHS) and hydrogen sulfide (H_2_S) donor GYY4137 on ischemic human myoblasts. To test the effect of NaHS, ischemic human myoblasts were treated with 1 mM, 100 μM, and 10 μM of NaHS for 20 min before the end of the 6 h hypoxia treatment and then for additional 3 h under the normoxia condition **(A,B)**. Similarly, ischemic human myoblasts were treated with 100 and 10 μM of GYY4137 for 24 h **(C,D)**. The effect of both compounds on cell proliferation and reversal of cell toxicity was determined by MTS and LDH assays, respectively. Data are expressed as mean ± SD (*n* = 6).

Since NaHS and GYY4137 could protect organs and tissues against Ischemia-reperfusion (I/R) injury, we here evaluated the effect of NaHS and GYY4137 on ischemic human myoblasts by MTS and LDH assays. The results of these experiments are shown in Figure 5. Compared to the control treatment with an exosome-free medium (i.e., phenol red-free DMEM supplemented with 1% exosome-depleted FBS), all tested concentrations of NaHS and GYY4137 were unable to significantly improve the hypoxia-induced ischemic injury to human myoblasts. These findings suggest that NaHS and GYY4137 are ineffective in ameliorating the ischemic injury compared to hADSC-derived exosomes obtained using the UF method, which showed increase viability and reduced toxicity to ischemic injury human myoblasts cells under the same conditions of the experiment (Figures 4A–D).

## Discussion

Research in EVs has recently received increasing attention; however, one major roadblock is the lack of a scalable technique permitting efficient purification of the small EVs (exosomes). Here we report the first systematic comparison study evaluating biochemical and functional aspects of UF- and UC-purified exosomes while also optimizing their preservation method. NTA and WB revealed that while exosomes isolated from hADSC cultures via both methods have similar size distributions, the UF/TFF method produces substantially higher yields of exosomes than the standard UC method. Moreover, exosomes obtained using the UF/TFF method and lyophilized in the presence of trehalose and PVP retained their size and numbers, as well as their bioactivity in terms of ameliorating the hypoxia-induced ischemic injury to human myoblasts.

Among the methodologies used for isolation of EVs and thus exosomes, the UC method has been remained as a gold standard and is widely used (Thery et al., 2018; Li et al., 2019). However, the UC method has some drawbacks such as low recovery rates, low yield, being highly time consuming, and changing the exosome’s morphology and composition due to high centrifugal forces (Lamparski et al., 2002; Witwer et al., 2013; Greening et al., 2015; Linares et al., 2015). In recent years, other approaches such as UF/TFF, size exclusion chromatography (SEC), and microfluidic platforms have been introduced (Boing et al., 2014; Gholizadeh et al., 2017; Busatto et al., 2018). Although SEC and microfluidic techniques are promising methods, they have also some disadvantages such as low capability and complex manufacturing processes. In the present study, we systematically compared the UC and UF/TFF methods. In our hands, the UF/TFF method proved to be more efficient in terms of exosome yield and bioactivity and allowed fast processing of large volumes.

In addition to a simple and efficient isolation method, exosome research also requires effective long-term preservation methods for realization of its therapeutic potential. Lyophilization would represent an ideal solution to the long-term storage challenge of exosomes. It may allow storing freeze-dried samples at room temperatures, and thus offers several advantages including addressing biosafety issues by preventing cross-contamination of samples (Tedder et al., 1995; Bielanski et al., 2000), facilitating sample transport, and greatly reducing storage and transport costs. However, lyophilization is a complex process involving both freezing and drying and applies considerable stresses to biological samples as a result of the removal of both unbound and bound water. Typically, completion of primary and secondary drying (complete lyophilization) results in 0.05 g H2O per g dry weight or lower (Zhang et al., 2017). At such low water content, lipid membranes usually undergo phase transition from a liquid crystalline to a gel-like state, which would lead to phase separation and a leaky membrane. Consequently, membrane-bound vesicles such as exosomes are expected to be leaky during the freeze-drying and rehydration process if not properly protected.

In nature, many organisms including tardigrades, some insects, brine shrimp, bacteria, and yeasts survive extreme drying, and thus address the phase separation issue and other drying-associated stresses mainly by accumulating intra- and extracellular sugars such as trehalose (Crowe et al., 1992; Potts, 1994). The protective effect of trehalose during drying and rehydration has also been demonstrated in different model systems (e.g., liposomes, membranes, and proteins) and mammalian cells (Crowe et al., 1993a, b, 1987, 1994; Remmele et al., 1997; Guo et al., 2000; Chen et al., 2001). As a non-reducing disaccharide, trehalose has a remarkably high glass transition temperatures (Tg) (Levine and Slade, 1988; Eroglu et al., 2009; Eroglu, 2010), which would support the resulting vitrified state at high temperatures. Therefore, trehalose has been extensively used for preservation of biological materials (Bissoyi et al., 2016). Experimental evidence suggest that trehalose exerts its protective effects (i) by stabilizing lipid membranes as a result of direct interactions with polar residues through hydrogen bonding (so-called “water replacement hypothesis”) (Crowe et al., 1993a, b, 1996); (ii) by its excellent glass forming properties (vitrification hypothesis) (Koster et al., 1994); (iii) by preventing aggregations of intracellular proteins (Tanaka et al., 2004, 2005); and (iv) by acting as osmolyte/protectant against osmotic (Somero, 1986; Singer and Lindquist, 1998), chemical (Sola-Penna et al., 1997), oxidative (Benaroudj et al., 2001; Herdeiro et al., 2006), and hypoxic stresses (Chen and Haddad, 2004). The ability to directly interact with polar groups is particularly important for lyophilization because by replacing the lost water molecules in the dehydrated state via hydrogen bonding to lipid head groups, trehalose prevents phase separation, and thus leakage of lipid membranes (Crowe et al., 1993a, b, 1996).

Unlike trehalose-like small sugar molecules, polymers such as PVP, dextran, and ficoll cannot directly interact with polar lipid head groups through hydrogen bonding (Crowe et al., 1998). Nevertheless, such polymers may improve the stability of freeze-dried products by increasing the product’s Tg. Indeed, our previous experiments clearly showed that supplementing trehalose solutions with polymers such as PVP and ficoll greatly increases Tg of the resulting mixtures upon drying (Drake et al., 2018). Considering that trehalose-PVP mixtures tested in the present study have no adverse effects on exosomes and such mixtures have a very high Tg at low water contents (Drake et al., 2018), the tested combinations of trehalose and PVP (particularly 100 mM trehalose + 5% PVP40) are expected to preserve lyophilized exosome samples at ambient temperature without any significant activity lost even if the storage temperature fluctuates. In fact, our preliminary data on the residual moisture content of the freeze dried samples along with published data (Drake et al., 2018) suggest a Tg over 70°C for 100 mM trehalose + 5% PVP.

Skeletal muscle ischemia and reperfusion injury are now recognized as one of the most common and important clinical problems. The clinical need for an effective musculoskeletal injury therapy is in a great demand. Therapeutic interventions that change the biochemical environment during the ischemic and/or reperfusion period may result in amelioration of subsequent cellular damage. Hydrogen sulfide (NaHS or its donor GYY4137) has been proposed in many studies as a therapy to prevent IR damage by reducing free radical-induced stress, promoting mitochondrial function, activating vascularization pathways, and reducing apoptosis. Therefore, NaHS treatment represented an alternative approach to assess the effectiveness of our exosome treatment. Our experiments using the same cells under the same experimental conditions revealed that the hADSC-derived exosome treatment is more effective than the NaHS treatment in terms of ameliorating the ischemic injury, further supporting clinical applications of exosome therapies.

## Conclusion

In conclusion, the results of the present study support our hypotheses that (1) UF/TFF is an efficient method for isolating high yield, bioactive exosomes and (2) freeze-drying in the presence of trehalose and PVP better retains the size, number, and bioactivity of hADSC-derived exosomes. These findings may facilitate therapeutic application of exosomes.

## Data Availability statement

The datasets generated for this study are available on request to the corresponding author.

## Author Contributions

MH and AE designed the experiments and co-wrote the manuscript. KE and MN performed the experiments and prepared the figures and manuscript. FO’B, YL, and SF provided input on experimental design, methods, and research strategy.

## Conflict of Interest

The authors declare that the research was conducted in the absence of any commercial or financial relationships that could be construed as a potential conflict of interest.

## Footnotes

1 www.augusta.edu/mcg/cba/emhisto/
